# Primary spinal epidural non‐Hodgkin's diffuse large B‐cell lymphoma: A case report

**DOI:** 10.1002/ccr3.3122

**Published:** 2020-09-21

**Authors:** Nishan Babu Pokhrel, Rohit Prasad, Sushil Paudel, Dinesh Kafle, Rohit Kumar Pokharel

**Affiliations:** ^1^ Department of Orthopaedics and Trauma Surgery Tribhuvan University Institute of Medicine Kathmandu Nepal

**Keywords:** decompression, diffuse large B‐cell lymphoma, epidural, excisional biopsy, non‐Hodgkin's lymphoma, primary spinal epidural lymphoma

## Abstract

Rare disease like primary spinal epidural diffuse large B‐cell lymphoma should be considered as a differential diagnosis in patients presenting with back pain and rapid neurological deterioration in the lower extremities.

## Graphical abstract

Rare diseases like primary spinal epidural diffuse large B‐cell lymphoma should be considered as a differential diagnosis in patients presenting with back pain and rapid neurological deterioration in the lower extremities.

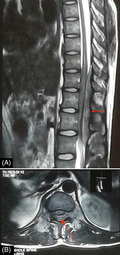

## 1 INTRODUCTION

A 24‐year‐old man presented with radiating back pain and progressive neurological deficit. MRI revealed a spinal epidural tumor at thoracolumbar junction. He underwent decompression and excisional biopsy. Histopathology and immunohistochemistry identified it as diffuse large B‐cell lymphoma. He received chemotherapy and is asymptomatic at 1‐year follow‐up.

Primary spinal epidural lymphomas (PSELs) comprise a group of tumors, which are present only in the spinal epidural space, with a histopathological picture of lymphoma and negative diagnostic workup for lymphoma at other sites.^1^ The epidural location of lymphomas, both Hodgkin's and non‐Hodgkin's, is infrequent and challenging to diagnose. About 24%‐48% of non‐Hodgkin's lymphomas (NHLs) were found to have an extranodal origin.^2^, ^3^ However, PSEL accounts for only 0.9% of all extranodal NHLs.^4^ There are many cell types of NHLs. Primary spinal epidural DLBCL is the most common form which accounts for only 1.8% all diffuse large B‐cell lymphomas.^5^ It is easily missed and maybe misdiagnosed leading to a lack of timely intervention and unhindered tumor progression.

This report describes the clinical features, imaging characteristics, and histopathological features of the rare case of primary spinal epidural diffuse large B‐cell lymphoma (DLBCL). To the best of our knowledge, this is the first reported case of primary spinal epidural DLBCL from Nepal. It also emphasizes the importance of a multidisciplinary approach for the successful treatment of this disease.

## 2 CASE REPORT

A 24‐year‐old man presented with a 3‐month history of low back pain associated with the burning sensation of bilateral lower limbs. For the last 12 days, he was unable to walk due to gradually progressive weakness in bilateral lower limbs. These symptoms were not accompanied by fever, headache, and night sweats. He could not recall any history of trauma in the past. His past medical and surgical history was not significant.

On examination, there was a localized tenderness over the thoracolumbar region. Lower limb motor power varied across muscle groups: hip flexors (2/5 bilaterally), knee extensors (2/5 bilaterally), ankle dorsiflexors (2/5 bilaterally), long toe extensors (3/5 bilaterally), and ankle plantar flexors (3/5 bilaterally). The sensation of lower limbs was altered bilaterally, but bowel and bladder habits were normal. Deep tendon reflexes in the knee and the ankle joints were absent. Plantar reflexes were downgoing bilaterally.

**FIGURE 1:**
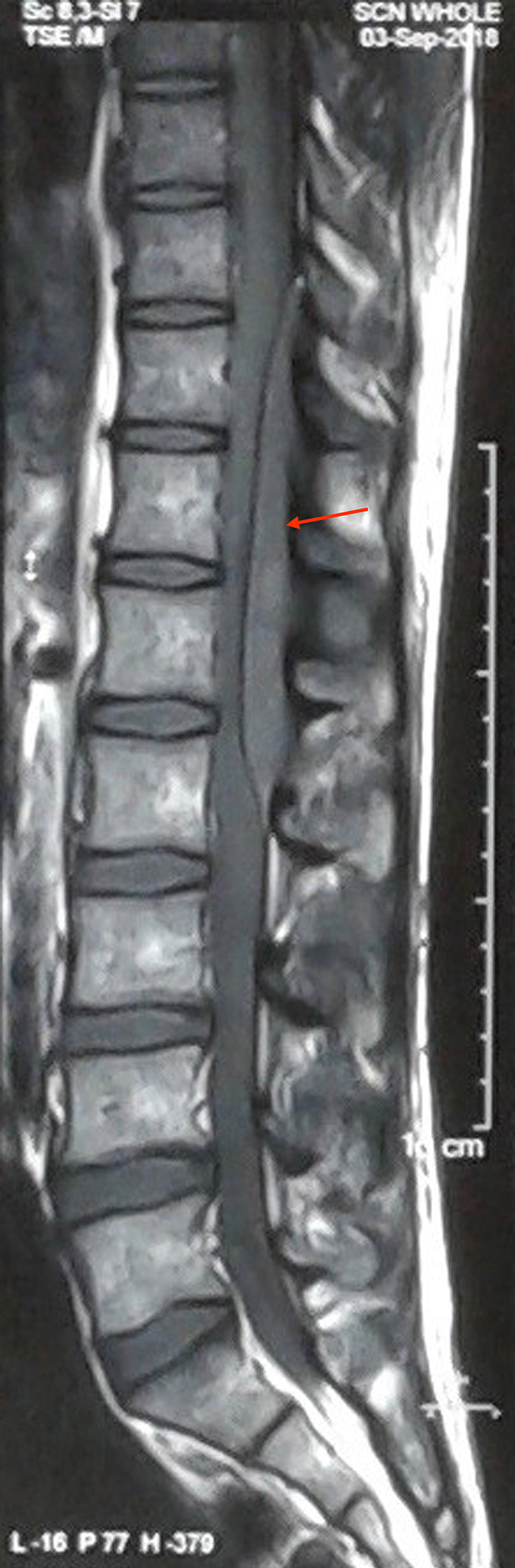
Sagittal T1W image shows well‐defined iso to hypointense lesion extending from the lower level of T10 vertebral body to L2 vertebral level in extramedullary region (red arrow). The lesion is severely compressing the spinal cord. The vertebral bodies show normal signal intensity. Intervertebral disk space is well maintained, and no prevertebral collection can be appreciated. T1W, T1 weighted.

**FIGURE 2:**
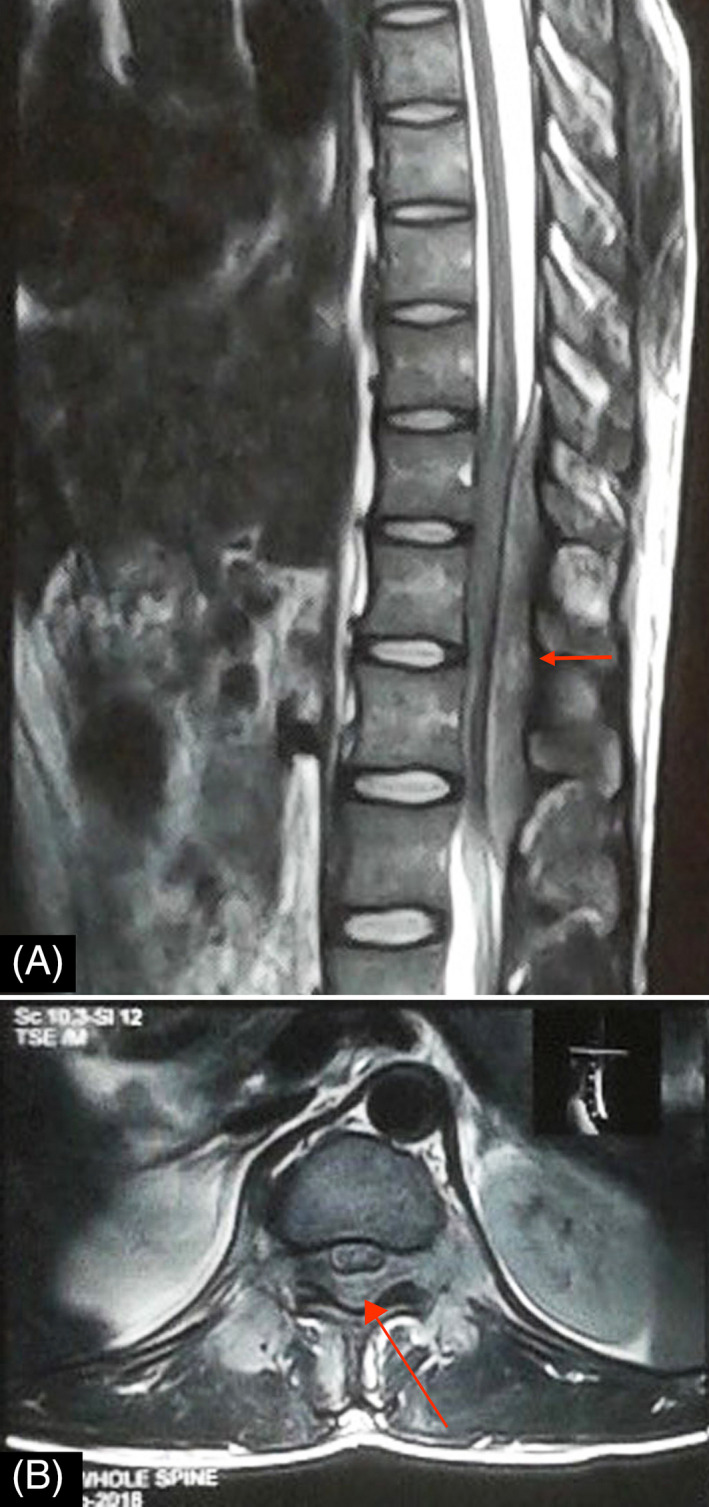
Sagittal (A) and axial (B) T2W images show well‐defined, heterogeneous, extramedullary, iso to hyperintense lesion (red arrows) extending from T10 to L2 vertebral levels, compressing, and displacing the spinal cord anteriorly. No obvious erosion of the vertebral body is seen. T2W, T2 weighted

**FIGURE 3:**
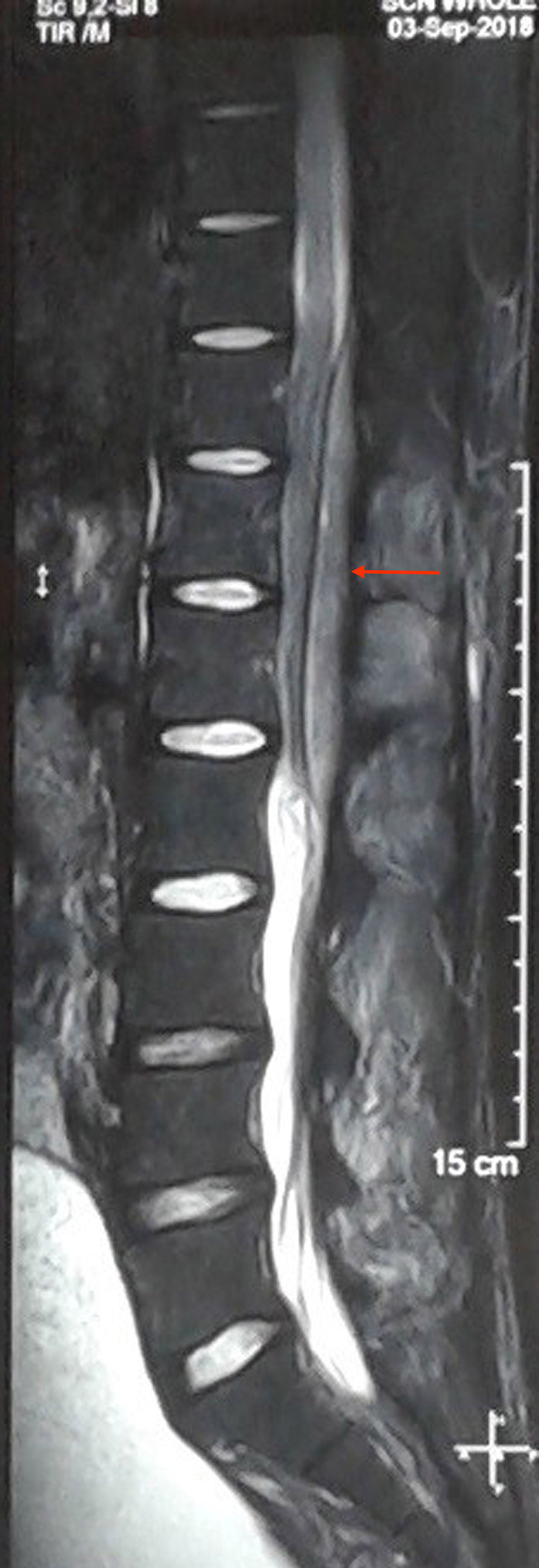
Sagittal STIR image showing extramedullary lesion (red arrow) extending from T10‐L2 levels with high signal intensity to the vertebral marrow which is compressing and displacing the spinal cord anteriorly. STIR, short tau inversion recovery

With the clinical diagnosis of a space‐occupying lesion in the thoracolumbar spine region, an X‐ray of dorsolumbar spine was done. Apart from the loss of lumbar lordosis, the rest of the X‐ray findings were normal. Magnetic resonance imaging (MRI) of the thoracolumbar spine revealed extradural lesion extending from T10 to L2 vertebral level. T1W image showed homogenous lesion, which was iso to hypointense and the T2W image showed heterogeneous lesion, which was iso to hyperintense, compressing over the spinal cord dorsally (Figures 1 and 2). Sagittal short tau inversion recovery (STIR) image also revealed an extramedullary lesion with high signal intensity to the vertebral marrow (Figure 3). Chest X‐ray, complete blood count (CBC), liver and renal function tests, erythrocyte sedimentation rate (ESR), c‐reactive protein (CRP), peripheral blood smear, lactate dehydrogenase (LDH), uric acid, and urinalysis were normal. The diagnosis of the extradural tumor over the T10‐L2 vertebrae was made.

**FIGURE 4:**
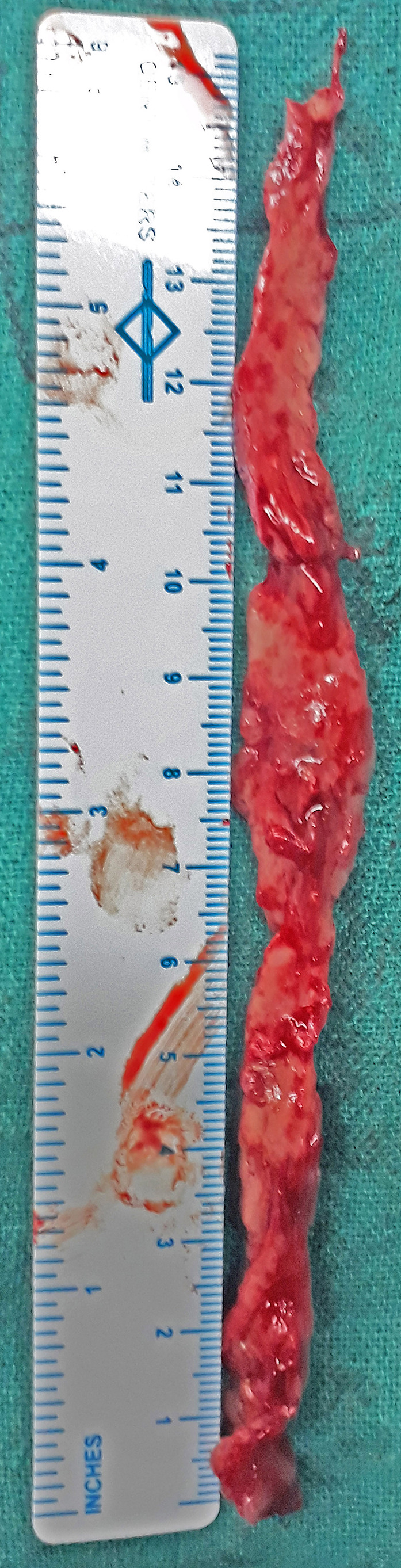
A 15 × 1 cm gray to white‐colored lesion which was soft to firm in consistency

**FIGURE 5:**
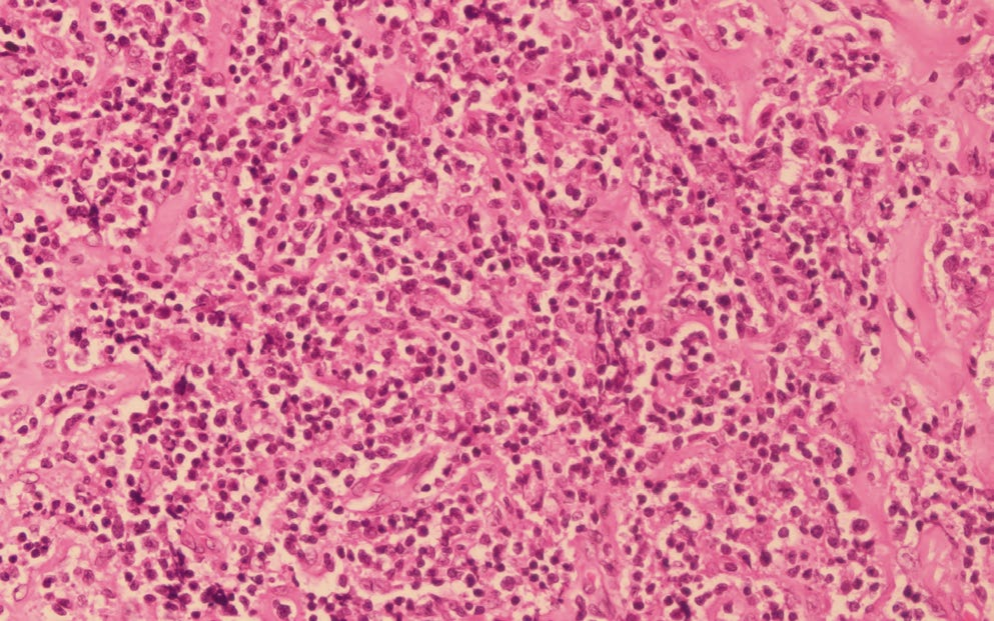
Histopathology (hematoxylin and eosin, ×200) revealing diffuse proliferation of intermediate‐sized atypical lymphoid cells infiltrating into the fibrocollagenous tissue. Few of the cells show nuclear irregularity with prominent nucleoli. Small lymphocytes are also seen in the adjacent area

Decompression (laminectomy) and excision biopsy of the mass were planned. The thoracolumbar spine (T9 to L3) was approached midline posteriorly. After laminectomy, a dorsally located greyish red extradural tumor extending from T10 to L2 was removed in strips. One of the long strips of the excised mass (approximately 15 × 1 cm) was sent for histopathological examination (Figure 4). Histopathology identified it as DLBCL (Figure 5). Immunohistochemistry analysis showed CD3^−^, CD20^+^, CD30^+^, CD10^+^, ALK‐1^−^, BCL‐6^+^, MUM‐1^−^, and Ki‐67^+^. No other lesion was detected on staging evaluation, which included contrast‐enhanced computed tomography (CECT) of chest, abdomen, and pelvis, and bone marrow aspiration and biopsy.

His postoperative period was uneventful with successful recovery in his neurological symptoms. He was referred to a cancer center after 16 days of hospital stay where he obtained six cycles of chemotherapy with rituximab, cyclophosphamide, doxorubicin, vincristine, and prednisolone (R‐CHOP) regimen. The interval between each cycle of chemotherapy was 3 weeks. Hematological parameters during and after the completion of chemotherapy were within normal limits. After the treatment, the tumor regressed, and he fully regained his muscle power. At the latest, 1‐year follow‐up, he is asymptomatic and is performing his routine daily activities.

## 3 DISCUSSION

Lymphomas are malignant tumors arising from lymphoid tissue. It can be Hodgkin's or non‐Hodgkin's type, later being more common, and their primary epidural occurrence is very rare.^4^ Out of all the primary central nervous system (CNS) tumors (which also includes spinal lymphomas), primary DLBCL of the CNS constitutes only 0.5% cases, occurring in approximately two cases per 10 million people. Spinal cord location is particularly rare.^6^

The pathogenesis of primary central nervous system lymphomas (PCNSLs) has been a matter of considerable debate among experts. Some authors mention that PCNSL arise de novo in nervous system tissues due to the presence of lymphoid precursor cells in this location.^7^, ^8^ Others argue that tumors from local and distant sites spread in the epidural space resulting in their apparent primary presentation.^9^ The remaining believe that an unrecognized pre‐existing retroperitoneal disease may exist before they present as PSEL.^10^

There is a male preponderance (69% vs 31%) of PSEL matching exactly with our case.^11^ Though the usual age of presentation is beyond 40 years of age, several cases have been reported in younger patients. Nambiar et al^12^ reported a case of primary thoracic (T5‐T10) spinal epidural B‐lymphoblastic lymphoma in a 19‐year‐old man from India. There are very few reported cases of spinal epidural NHL in younger age groups.^13^ Our case of a 24‐year‐old man is another addition to it.

The thoracic spine is the most commonly involved site, followed by the lumbosacral and cervical spine.^8^ The common occurrence of PSEL in the thoracic spine can be explained by the rich venous plexus in this site, greater length of the thoracic spine as compared with cervical and lumbar, more tolerance for bulky disease in the thorax and abdomen and the existence of an unrecognized preexisting retroperitoneal disease.^10^, ^14^ The usual location of the tumor in spinal epidural space is usually dorsal.^15^ Our case too had a similar finding of lesion present dorsally and located at the thoracolumbar region.

The onset of symptoms is usually subacute, occurring over a few days to weeks. The symptoms and signs of the disease depend on the location and size of the tumor. Localized back pain followed by lower limb weakness is the most common symptom.^11^ Local back pain, sometimes accompanied by radicular pain to legs and abdomen, can be the prodromal symptom that can persist for several months to a year. Then, the phase of rapid neurological deterioration occurs over 2‐8 weeks, which is due to spinal cord compression.^14^ Bladder and bowel disturbances appear only later in the course.^16^ It may give us the idea of time since the onset of the pathology. In our case, the presenting features were back pain radiating to bilateral lower limbs for 3 months followed by neurological deficits for the last 12 days without bladder involvement.

B symptoms (fever, night sweats, and weight loss) were rare, and none had hepatomegaly or splenomegaly in one of the largest surveys, which is consistent with our case. As most of the patients presented in the earlier stage, B symptoms were probably absent.^11^

MRI is the investigation of choice in the preoperative and postoperative evaluations.^10^ PSEL appears in MRI as isointense on T1W image and iso‐ to hyperintense on T2W image, with marked contrast enhancement.^15^ Contrast enhancement with Gadolinium further helps in the assessment of extraosseous soft tissue components. This allows a better differentiation of various pathologies (eg, metastatic carcinoma and sarcoma), tumor extent, and bony involvement.^15^ Similar features were recognized in our case too.

The whole‐body scan should be performed to identify lungs, pleural, splenic, gastric, intestinal, pancreatic, and renal involvement. Bone marrow biopsy from the sternum or iliac crest is required to rule out lymphoreticular involvement. CBC, LDH, and chest X‐ray should be performed as well. In our case, we could not find any positive reports. Lumbar puncture is not recommended, because it is potentially hazardous because of the coning phenomenon and it is also of limited diagnostic value as it shows a non‐specific increase in protein levels; neoplastic cells are rarely found.^17^, ^18^

Monnard et al^11^ have recommended surgical decompression in the form of partial or total removal of the tumor mass and/or decompressive laminectomy. Doing so, relieves the spinal cord compression and also establishes the correct histological diagnosis. Surgery is clearly indicated when the diagnosis is not yet established.^1^ Emergency decompression is needed only in cases of acute paresis and/or loss of bowel/bladder control.^16^ Functional recovery of patients suffering spinal cord compression due to PSEL is relatively better than that of patients with metastatic carcinoma.^4^

Histopathological examination of the biopsy specimen usually shows diffuse proliferation of atypical lymphoid cells,^8^ which is similar to our finding. Immunohistochemistry analysis with positive CD20^+^, CD10^+^, BCL6^+^, and MUM‐1^−^ further supported our diagnosis of DLBCL (germinal center type).^5^, ^19^ Positive Ki‐67 suggested the aggressiveness of the tumor.

Combined modality treatment, including radiotherapy and chemotherapy, has been recommended to be the most efficient treatment because lymphomas are very chemo‐ and radiosensitive tumors.^11^, ^18^, ^20^ In the largest survey until now, local control of 88% and 5‐year overall survival of 69% have been observed with combined modality treatment.^11^ Earlier diagnosis and treatment is associated with improved functional outcomes. Several authors report that patients with more aggressive histological tumor types have a poorer prognosis. Patients of over 40 years of age with aggressive histological tumors have a poor prognosis.^10^ Our case was a 24‐year‐old man, otherwise healthy, and was diagnosed early and underwent surgery with significant neurological improvement, which is the most favorable prognostic indicator of overall survival.^11^ He responded well to chemotherapy with complete neurological recovery.

## 4 CONCLUSION

Primary spinal epidural DLBCL is one of the rarest tumors due to which its diagnosis is challenging. Signs and symptoms are similar to those of any other epidural space‐occupying lesions and, thus, can be frequently misdiagnosed. Back pain with sudden neurological deterioration in lower limbs should raise the suspicion of rarer diseases like primary spinal epidural DLBCL. A high degree of suspicion and a team approach involving multiple specialties (neurosurgeon, medical oncologist, radiation oncologist, and pathologist) is vital for diagnosis and management.

## CONFLICT OF INTEREST

None declared.

## AUTHOR CONTRIBUTIONS

NBP, RP, and SP: wrote the initial draft, edited it, and reshaped into this manuscript. DK and RKP: reviewed the manuscript. All authors: approved the final version of the manuscript and agreed to be accountable for all aspects of the work in ensuring that questions related to the accuracy or integrity of any part of the work are appropriately investigated and resolved.

## ETHICS APPROVAL AND CONSENT TO PARTICIPATE

Need for ethical approval waived. Consent from the patient deemed to be enough.

## CONSENT FOR PUBLICATION

Written informed consent was obtained from the patient for publication of this case report and any accompanying images. A copy of the written consent is available for review by the editor‐in‐chief of this journal.

## Data Availability

Not applicable.
